# Clinical value of fluorine-18α-methyltyrosine PET in patients with gliomas: comparison with fluorine-18 fluorodeoxyglucose PET

**DOI:** 10.1186/s13550-017-0298-8

**Published:** 2017-05-31

**Authors:** Keishi Horiguchi, Masahiko Tosaka, Tetsuya Higuchi, Yukiko Arisaka, Kenichi Sugawara, Junko Hirato, Hideaki Yokoo, Yoshito Tsushima, Yuhei Yoshimoto

**Affiliations:** 1 Department of Neurosurgery, Gunma University Graduate School of Medicine, 3-39-22 Showa-machi, Maebashi, Gunma 371-8511 Japan; 2 Department of Diagnostic Radiology and Nuclear Medicine, Gunma University Graduate School of Medicine, Maebashi, Gunma Japan; 3 Department of Human Pathology, Gunma University Graduate School of Medicine, Maebashi, Gunma Japan; 4 Department of Pathology, Gunma University Hospital, Maebashi, Gunma Japan

**Keywords:** ^18^F-FAMT, PET, Glioma, ^18^F-FDG, MIB-1 labeling index

## Abstract

**Background:**

We investigated the relationship between metabolic activity and histological features of gliomas using fluorine-18α-methyltyrosine (^18^F-FAMT) positron emission tomography (PET) compared with fluorine-18 fluorodeoxyglucose (^18^F-FDG) PET in 38 consecutive glioma patients. The tumor to normal brain ratios (T/N ratios) were calculated, and the relationships between T/N ratio and World Health Organization tumor grade or MIB-1 labeling index were evaluated. The diagnostic values of T/N ratios were assessed using receiver operating characteristic (ROC) curve analyses to differentiate between high-grade gliomas (HGGs) and low-grade gliomas (LGGs).

**Results:**

Median T/N ratio of ^18^F-FAMT PET was 2.85, 4.65, and 4.09 for grade II, III, and IV gliomas, respectively, with significant differences between HGGs and LGGs (*p* = 0.006). Both T/N ratio (*p* = 0.016) and maximum standardized uptake value (*p* = 0.033) of ^18^F-FDG PET showed significant differences between HGGs and LGGs. ROC analysis yielded an optimal cut-off of 3.37 for the T/N ratio of ^18^F-FAMT PET to differentiate between HGGs and LGGs (sensitivity 81%, specificity 67%, accuracy 76%, area under the ROC curve 0.776). Positive predictive value was 84%, and negative predictive value was 62%. T/N ratio of ^18^F-FAMT PET was not correlated with MIB-1 labeling index in all gliomas, whereas T/N ratio of ^18^F-FDG PET was positively correlated (*r*_*s*_ = 0.400, *p* = 0.013). Significant positive correlation was observed between T/N ratios of ^18^F-FDG and ^18^F-FAMT (*r*_*s*_ = 0.454, *p* = 0.004), but median T/N ratio of ^18^F-FAMT PET was significantly higher than that of ^18^F-FDG PET in all grades of glioma.

**Conclusions:**

The T/N ratio of ^18^F-FAMT uptake has high positive predictive value for detection of HGGs. ^18^F-FAMT PET had higher T/N ratio, with better tumor-normal brain contrast, compared to ^18^F-FDG PET in both LGGs and HGGs. Therefore, ^18^F-FAMT is a useful radiotracer for the preoperative visualization of gliomas.

## Background

Magnetic resonance imaging with or without gadolinium enhancement is the standard method for the diagnosis of brain tumors, but new imaging methods have also been proposed based on the specific metabolic characteristics of gliomas. Malignant gliomas have increased metabolism caused by anaerobic glycolysis, so that positron emission tomography (PET) using fluorine-18 fluorodeoxyglucose (^18^F-FDG), a glucose analog, is now widely used for the diagnosis of gliomas [1]. However, the high utilization of glucose by normal gray matter makes identification of other brain tumors difficult on ^18^F-FDG PET [2]. Consequently, PET imaging of glucose metabolism is basically unsuitable for the detection of tumors against the background of the normal brain.

Radiolabeled amino acids are well-established tracers for brain tumor imaging with PET. The Response Assessment in Neuro-Oncology working group has recently recommended the use of amino acid PET imaging for brain tumor management in addition to magnetic resonance imaging [3, 4]. L-[methyl-^11^C]methionine (^11^C-MET) is the most widely used amino acid PET imaging tracer for gliomas for the preoperative detection, diagnosis of subtypes and grades, differential diagnosis from radiation necrosis, estimation of tumor infiltration, and delineation of the border of tumor removal [5, 6]. Methyl-^11^C-choline, another PET radiotracer, potentially reflects the grade of malignancy [7]. However, the short half-life (20 min) of ^11^C requires in-house radiosynthesis and repeated radiolabeling of the tracer for each PET study, resulting in limited use only in PET centers with an in-house cyclotron facility [7]. Consequently, development of an amino acid tracer using the long half-life of ^18^F has been desirable to overcome these disadvantages of ^11^C-labeled agents [2]. Recently, the ^18^F-based PET tracers, *O*-(2-[^18^F]fluoroethyl)-L-tyrosine (^18^F-FET) and L-6-[^18^F]fluoro-3,4-dihydroxyphenylalnine have been used for the imaging of brain tumors [8–12]. In Europe, the high clinical interest in ^18^F-FET PET has led to more than 10000 PET scans being performed in some centers [13].

Previously, we developed L-[3-^18^F]-α-methyltyrosine (^18^F-FAMT), a new amino acid tracer for PET imaging and demonstrated its potential for detecting neoplasms using experimental tumor models [14, 15]. ^18^F-FAMT accumulates in tumor cells only via an amino acid transport system, and most of the incorporated ^18^F-FAMT is not metabolized [14, 15]. Recently, we have made advances in the clinical utility of ^18^F-FAMT PET for the investigation of lung cancers, oral and maxillofacial cancers, and other tumors [16–20]. Our preliminary study showed specific accumulation of the tracer in 15 patients with glioma, including 7 cases before treatment [21]. However, no detailed study has assessed ^18^F-FAMT PET in a glioma series.

The present study investigated the value of ^18^F-FAMT uptake for differentiating high-grade glioma (HGG) from low-grade glioma (LGG) and the correlation with the proliferation rate, compared with ^18^F-FDG as the standard PET tracer.

## Methods

### Patients

The clinical records of patients treated between July 2007 and December 2013 were retrospectively reviewed. The criteria for inclusion were (i) histopathology of the tumor was established by open surgery or by stereotactic biopsy, and (ii) both ^18^F-FAMT PET and ^18^F-FDG PET were performed in random order before surgery within 2 months. The histological type of the tumors was determined by the World Health Organization (WHO) classification system [22]. No cases of pilocytic astrocytomas (WHO grade I) were included. Extremely rare histological types were also excluded. None of the patients had insulin-dependent diabetes, and serum glucose levels were less than 120 mg/dL in all patients just before ^18^F-FAMT or ^18^F-FDG injection. All patients agreed to participate in this study and provided written informed consent. This study was approved by the institutional review board of Gunma University Graduate School of Medicine.

### PET studies

Both ^18^F-FDG and ^18^F-FAMT were synthesized in the cyclotron facility of our institute, with ^18^F-FAMT produced according to the methods of Tomiyoshi et al. [14].

In this study, PET used a Discovery STE (GE Healthcare, Waukesha, WI, USA) or Biograph 16 (Siemens Medical Solutions, Knoxville, TN, USA) scanner, with field of view of 700 mm and slice thickness of 3.27 mm. Three-dimensional data acquisition was performed for 3 min per bed position, followed by imaging reconstruction with the three-dimensional ordered-subset expectation maximization method. Correction of segmented attenuation was based on 128 × 128 matrix images obtained by X-ray computed tomography (CT) (140 kV, 120–240 mAs) without intravenous contrast material. CT images were reconstructed using a conventional filtered back projection method. Axial full-width half-maximum at 1 cm from the center of field of view was 5.6 mm, and z-axis full-width half-maximum at 1 cm from the center of field of view was 6.3 mm. Intrinsic system sensitivity was 8.5 cps/kBq for three-dimensional acquisition. Patients were scanned from the thigh to the head in the arms-down position. Limited breath-holding at normal expiration was used during CT to avoid motion-induced artifacts and allow co-registration of CT and PET images in the area of the diaphragm.

### Imaging analysis

All ^18^F-FAMT PET and ^18^F-FDG PET images were interpreted by two independent experienced nuclear physicians (Y.A. and T.H.), and final values were determined by consensus. Spearman’s correlation coefficient was used to evaluate the interrater reliability. The interpreting physicians were unaware of the patient’s clinical history and data. For semi-quantitative analysis, the standardized uptake value (SUV) was obtained from an attenuation-corrected transaxial image. SUV was calculated as follows: radioactive concentration in the region of interest (ROI) (MBq/g)/injected dose (MBq)/patient body weight (g). The ROI was manually drawn over the primary tumor. ROI analysis was conducted by a nuclear medicine physician with reference to the CT and magnetic resonance images. The maximum SUV (SUV_max_) and mean SUV in the ROI represent the uptake of ^18^F-FAMT and ^18^F-FDG in the lesion [23]. The ratio of the SUV_max_ in the tumor to the mean SUV in the contralateral normal brain (T/N ratio) was calculated. If the lesion was located in the thalamus, the T/N ratio to the contralateral normal thalamus was calculated.

### Histological study

Surgical or biopsy specimens were fixed in 10% formalin and were embedded in paraffin. The histological tumor type was established based on specimens stained with hematoxylin and eosin, according to the previous WHO criteria [22]. The relevance of this investigation is limited by the fact that the tumor classification is based on the previous, out-of-date WHO classification and so may no longer be directly applicable to the current classification [24].

The cellular proliferation activity of the tumor was determined by measuring the MIB-1 proliferation index obtained by immunohistochemical staining with anti-Ki-67/MIB-1 antibody (Dako, Tokyo, Japan). The percentage of tumor cells stained positively for MIB-1 antigen (MIB-1 labeling index: MIB-1 LI) was determined in the area containing the largest number of positive tumor cells and was regarded as representative of the tumor proliferation activity.

### Statistical analysis

All values are reported as proportions (%) or medians with interquartile range. Between-group comparisons of non-parametric data were performed using the Mann-Whitney *U* test. To compare T/N ratios between ^18^F-FAMT and ^18^F-FDG, the Wilcoxon’s signed-rank test was used because of matched pairing. The correlation between different variables was analyzed using the non-parametric Spearman’s rank test. Probability values of <0.05 indicated a statistically significant difference. The diagnostic accuracy of the T/N ratio and SUV_max_ of ^18^F-FAMT and ^18^F-FDG uptake for differentiation of HGGs from LGGs was evaluated by receiver-operating-characteristic (ROC) curve analysis using subsequent histological analysis of all lesions. The decision cutoff was considered optimal at the maximum of the product of paired values for sensitivity and specificity. In addition, the area under the ROC curve (AUC), its median, and the level of significance were determined as measures of the diagnostic quality of the test. For ROC analysis, the gliomas of WHO grades III and IV were considered together as HGGs. Statistical analysis was performed using SPSS version 21 (IBM Corp., Armonk, NY, USA) for Mac.

## Results

### Uptake of ^18^F-FAMT and ^18^F-FDG

This study included 38 patients (24 men and 14 women), aged 16 to 79 years (median 52.5 years). The final pathological diagnosis was based on samples obtained by open craniotomy (*n* = 37) or biopsy (*n* = 1). The tumors were classified as WHO grade II in 12 patients, grade III in 12, and grade IV in 14, and the histological diagnoses are summarized in Table 1. The median SUV in the contralateral normal cortex was 0.94 (range 0.49 to 1.50) for ^18^F-FAMT, and 5.91 (range 2.20–18.9) for ^18^F-FDG. The SUV_max_ and T/N ratio of ^18^F-FAMT and ^18^F-FDG are summarized in Table 2. Correlation coefficient and interobserver agreement for quantitative measurements was very high in all cases (*p* < 0.01).

**Table 1:** Histological characteristics of the tumors

Histopathology	*n* (%)	Median MIB-1 LI (IQR)
WHO grade II	12 (32)	4.1 (2.9–7.6)
Diffuse astrocytoma	3 (8)	7.4
Oligoastrocytoma	7 (18)	3.8 (2.7–4.4)
Oligodendroglioma	2 (5)	5.3
WHO grade III	12 (32)	16 (12–27)
Anaplastic astrocytoma	3 (8)	30
Anaplastic oligoastrocytoma	5 (13)	14 (9–21)
Anaplastic oligodendroglioma	4 (11)	16 (7.7–19)
WHO grade IV	14 (37)	28 (21–41)
Glioblastoma	14 (37)	28 (21–41)

*MIB-1 LI* MIB-1 labeling index, *IQR* interquartile range

**Table 2:** ^18^F-FAMT and ^18^F-FDG uptake in various tumor types

Histological classification	^18^F-FAMT		^18^F-FDG	
	Median SUV_max_ (IQR)	Median T/N ratio (IQR)	Median SUV_max_ (IQR)	Median T/N ratio (IQR)
All gliomas	3.45 (2.58–4.63)	4.08 (2.87–4.76)	6.65 (5.55–10.2)	1.12 (0.86–1.83)
WHO grade II	2.88 (2.04–3.75)	2.85 (2.06–4.28)	5.40 (4.20–8.45)	0.88 (0.75–1.04)
Diffuse astrocytoma	3.07	2.05	4.10	0.90
Oligoastrocytoma	2.65 (1.90–4.00)	2.80 (2.11–4.33)	5.10 (4.50–5.80)	0.86 (0.62–0.88)
Oligodendroglioma	3.60	4.23	10.7	1.39
WHO grade III	4.20 (2.17–5.75)	4.65 (3.50–5.42)	6.55 (6.00–8.28)	1.17 (0.68–2.24)
Anaplastic astrocytoma	2.63	5.20	6.60	1.22
Anaplastic oligoastrocytoma	2.68 (1.61–5.15)	4.29 (2.47–5.05)	6.00 (4.93–6.45)	0.71 (0.63–1.17)
Anaplastic oligodendroglioma	5.70 (4.93–6.40)	5.03 (3.71–6.39)	12.8 (6.70–21.3)	1.91 (1.17–2.75)
WHO grade IV	3.60 (3.05–4.60)	4.09 (3.36–4.92)	9.15 (6.38–11.5)	1.56 (1.11–2.50)
Glioblastoma	3.60 (3.05–4.60)	4.09 (3.36–4.92)	9.15 (6.38–11.5)	1.56 (1.11–2.50)

*IQR* interquartile range

### T/N ratio and SUV_max_

**Fig. 1:**
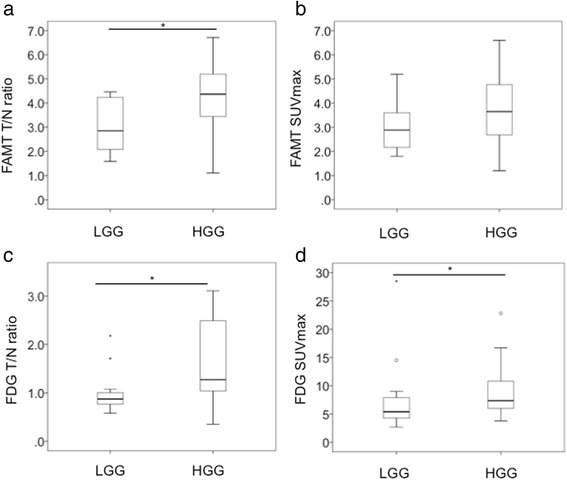
Box-and-whisker plots of ^18^F-FAMT and ^8^F-FDG uptake for LGG and HGG. **a** The difference in T/N ratio of ^18^F-FAMT uptake between LGG and HGG was significant (*p* = 0.006). **b** The difference in SUV_max_ of ^18^F-FAMT uptake between LGG and HGG was not significant (*p* = 0.087). **c**, **d** The differences in T/N ratio and SUV_max_ of ^18^F-FDG uptake between LGG and HGG were significant (*p* = 0.016 and *p* = 0.033, respectively)

The median T/N ratio of ^18^F-FAMT PET was 2.85, 4.65, and 4.09 for grades II, III, and IV in all gliomas, respectively (Table 2). The median T/N ratio of ^18^F-FAMT PET was 2.85 for LGGs and 4.37 for HGGs, showing a significant difference between LGGs and HGGs (*p* = 0.006, Fig. 1a). The median SUV_max_ of ^18^F-FAMT PET was 2.88, 4.20, and 3.60 for grade II, III, and IV gliomas, respectively. However, the SUV_max_ of ^18^F-FAMT PET showed no significant differences between LGGs and HGGs (*p* = 0.087, Fig. 1b).

The median T/N ratio of ^18^F-FDG PET was 0.88, 1.17, and 1.56 for grade II, III, and IV gliomas, respectively (Table 2). The median T/N ratio of ^18^F-FDG PET was 0.88 for LGGs and 1.27 for HGGs. The median SUV_max_ of ^18^F-FDG PET was 5.40, 6.55, and 9.15 for grade II, III, and IV gliomas, respectively. The median SUV_max_ of ^18^F-FDG PET was 5.40 for LGGs and 7.37 for HGGs. Both the T/N ratio and the SUV_max_ of ^18^F-FDG PET showed significant differences between HGGs and LGGs (*p* = 0.016 and *p* = 0.033, respectively, Fig. 1c, d).

### ROC analysis for ^18^F-FAMT PET and ^18^F-FDG PET

**Fig. 2:**
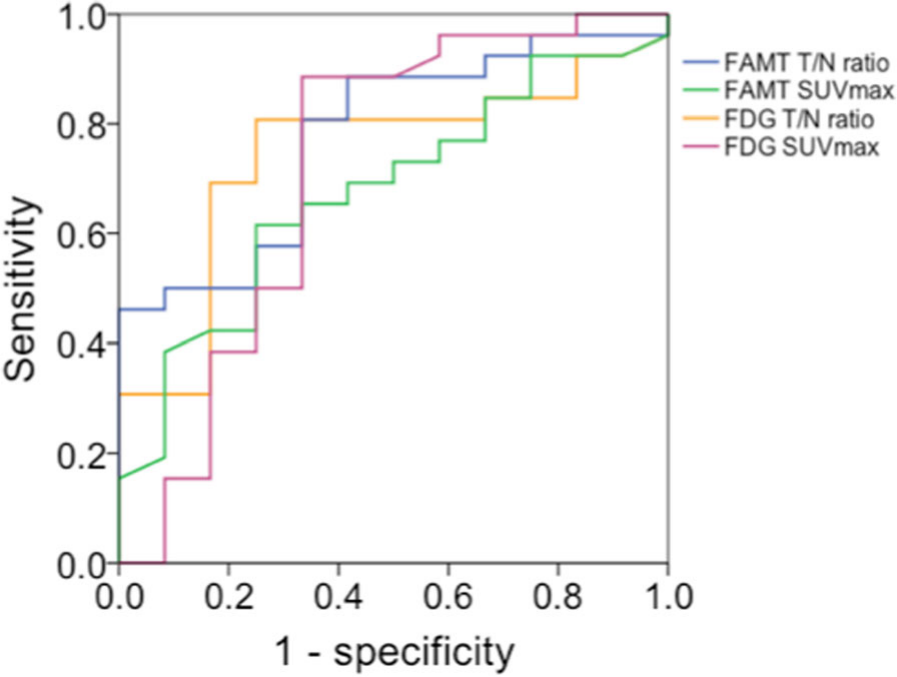
ROC curve analysis of T/N ratio (*blue curve*) and SUV_max_ (*green curve*) of ^8^F-FAMT to differentiate between HGGs and LGGs. Area under the curve was 0.776 for T/N ratio (*p* = 0.007, 95% CI 0.623–0.928, optimal cutoff 3.37) and 0.675 for SUV_max_ (*p* = 0.087, 95% CI 0.497–0.852, optimal cutoff 3.45). ROC curve analysis of T/N ratio (*orange curve*) and SUV_max_ (*purple curve*) of ^8^F-FDG to differentiate between HGGs and LGGs. Area under the curve was 0.742 for T/N ratio (*p* = 0.018, 95% CI 0.575–0.909, optimal cutoff 0.92) and 0.716 for SUV_max_ (*p* = 0.034, 95% CI 0.510–0.923, optimal cutoff 5.85)

T/N ratio of ^18^F-FAMT PET was significantly higher for HGGs (*n* = 26) than for LGGs (*n* = 12) (4.37 ± 2.22 vs. 2.85 ± 1.81; *p* = 0.006) (Fig. 1a). ROC analysis for differentiation between HGGs and LGGs yielded an optimal cutoff of 3.37 for the T/N ratio (sensitivity 81%, specificity 67%, accuracy 76%, AUC 0.776, 95% confidence interval [CI] 0.623–0.928). The positive predictive value (PPV) was 84%, and the negative predictive value (NPV) was 62% (Fig. 2). On the other hand, the SUV_max_ of ^18^F-FAMT PET showed no significant differences between HGGs and LGGs (3.65 ± 2.16 vs. 2.88 ± 1.71; *p* = 0.087) (Fig. 1b). ROC analysis for differentiation between HGGs and LGGs yielded an optimal cutoff of 3.45 for SUV_max_ of ^18^F-FAMT PET (sensitivity 61.5%, specificity 75%, accuracy 66%, AUC 0.675, 95% CI 0.497–0.852). The PPV was 84%, and the NPV was 47% (Fig. 2).

T/N ratio of ^18^F-FDG PET was significantly higher for HGGs than for LGGs (1.27 ± 1.47 vs. 0.88 ± 0.29; *p* = 0.016) (Fig. 1c). ROC analysis yielded an optimal cutoff of 0.92 for the T/N ratio of ^18^F-FDG PET to differentiate between HGGs and LGGs (sensitivity 81%, specificity 67%, accuracy 76%, AUC 0.742, 95% CI 0.575–0.909). The PPV was 84%, and the NPV was 62% (Fig. 2). Similarly, SUV_max_ of ^18^F-FDG PET was significantly higher for HGGs than for LGGs (7.37 ± 4.92 vs. 5.40 ± 4.25; *p* = 0.033) (Fig. 1d). ROC analysis for differentiation between HGGs and LGGs yielded an optimal cutoff of 5.85 for SUV_max_ of ^18^F-FDG PET (sensitivity 89%, specificity 67%, accuracy 82, AUC 0.716, 95% CI 0.510–0.923). The PPV was 85%, and the NPV was 73% (Fig. 2).

### MIB-1 LI and T/N ratio

**Fig. 3:**
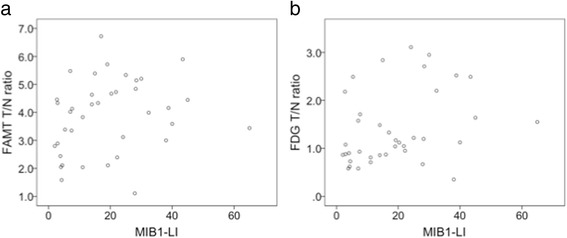
Correlation analysis between the MIB-1 LI and the T/N ratio of ^18^F-FAMT PET or ^18^F-FDG PET. **a** The T/N ratio of ^18^F-FAMT PET was not correlated with MIB-1 LI in all gliomas (*r*_*s*_ = 0.290, *p* = 0.077). **b** The T/N ratio of ^18^F-FDG PET was positively correlated with MIB-1 LI in all gliomas (*r*_*s*_ = 0.400, *p* = 0.013)

Neither the SUV_max_ nor the T/N ratio of ^18^F-FAMT PET was correlated with the MIB-1 LI in all gliomas (SUV_max_: *r*_*s*_ = 0.138, *p* = 0.408; T/N ratio: *r*_*s*_ = 0.290, *p* = 0.077; Fig. 3a). The T/N ratio of ^18^F-FDG PET was also positively correlated with the MIB-1 LI in all gliomas (*r*_*s*_ = 0.400, *p* = 0.013; Fig. 3b), whereas the SUV_max_ of ^18^F-FDG PET was not correlated (*r*_*s*_ = 0.242, *p* = 0.144).

### Comparison of ^18^F-FAMT and ^18^F-FDG

**Fig. 4:**
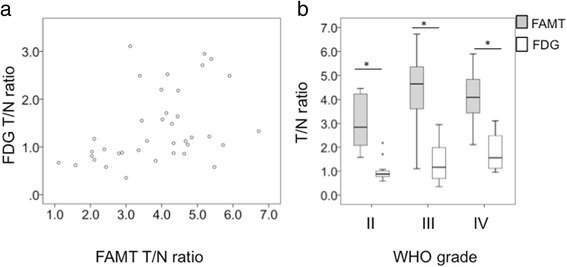
**a** Correlation analysis between the T/N ratios of ^18^F-FAMT PET and ^18^F-FDG PET showed significant positive correlation in all gliomas (*r*_*s*_ = 0.430, *p* < 0.01). **b** Comparison of the T/N ratios of ^18^F-FAMT PET and ^18^F-FDG PET found significant differences for each WHO grade (all *p* < 0.05)

**Fig. 5:**
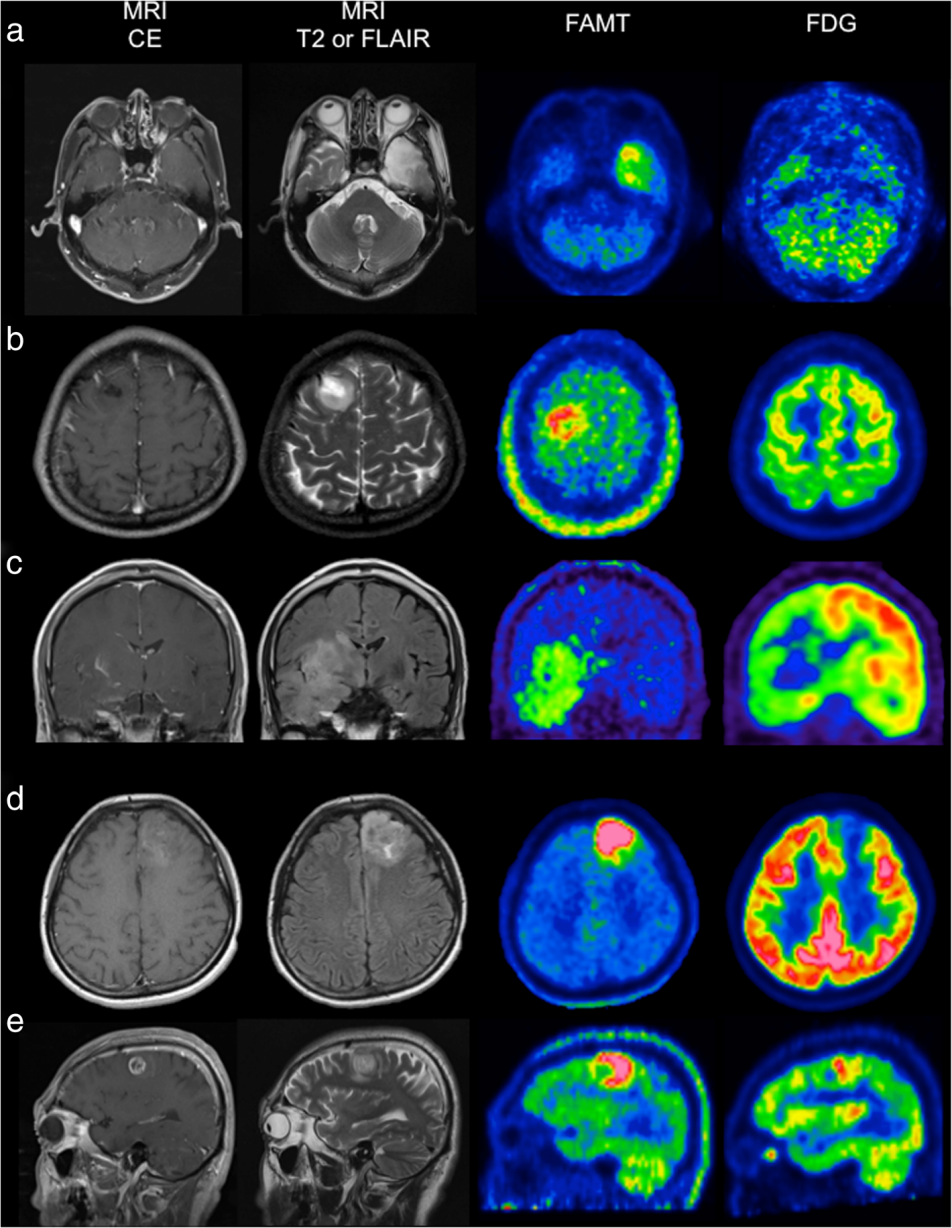
Representative cases. Contrast-enhanced T1-weighted magnetic resonance images (*MRI CE*), T2-weighted or fluid attenuated inversion recovery images (*MRI T2 or FLAIR*), ^18^F-FAMT PET images, and ^18^F-FDG PET images, *from left to right*. **a** A 71-year-old man with diffuse astrocytoma. ^18^F-FAMT T/N ratio was 2.05. ^18^F-FDG T/N ratio was 0.90. **b** A 32-year-old man with oligoastrocytoma. ^18^F-FAMT T/N ratio was 2.80. ^18^F-FDG T/N ratio was 0.86. **c** A 50-year-old man with anaplastic astrocytoma. ^18^F-FAMT T/N ratio was 5.33. ^18^F-FDG T/N ratio was 1.22. **d** A 39-year-old woman with anaplastic oligoastrocytoma. ^18^F-FAMT T/N ratio was 5.48. ^18^F-FDG T/N ratio was 0.58. **e** A 56-year-old man with glioblastoma. ^18^F-FAMT T/N ratio was 4.73. ^18^F-FDG T/N ratio was 1.05

Significant positive correlation was observed between the T/N ratios of ^18^F-FDG and ^18^F-FAMT in all gliomas (*r*_*s*_ = 0.454, *p* = 0.004; Fig. 4a). The median T/N ratio of ^18^F-FAMT was significantly higher than that of ^18^F-FDG in all gliomas (*p* < 0.05; Fig. 4b). The T/N ratio of ^18^F-FDG was lower than 1.0 in 15 (39%) of 38 gliomas, resulting in poor tumor-normal brain contrast. On the other hand, the T/N ratio of ^18^F-FAMT was greater than 2.0 in 36 (95%) of 38 gliomas, and all cases showed clear tumor-normal brain contrast. These results indicated that ^18^F-FAMT provides better tumor-normal brain contrast. Representative cases are shown in Fig. 5.

## Discussion

The T/N ratios of both ^18^F-FAMT and ^18^F-FDG were significantly higher for HGGs than for LGGs, although the T/N ratios of different tumor grades showed wide overlap. For HGGs, ^18^F-FAMT uptake beyond a T/N ratio cutoff of 3.37 or ^18^F-FDG uptake beyond a T/N ratio cutoff of 0.92 had a PPV of 84 or 84%, respectively. The T/N ratios of ^18^F-FAMT were not correlated with MIB-1 LI in all gliomas. The T/N ratio of ^18^F-FAMT was significantly higher than that of ^18^F-FDG in all gliomas and all tumor subtypes.

Radiosynthesis of ^18^F-FAMT, an amino acid analog with a relatively high chemical yield, was originally developed at our institute [14], and experimental and clinical investigations have demonstrated that accumulation of ^18^F-FAMT in tumor cells occurs via an amino acid transport system [14, 15, 23]. ^18^F-FAMT was predicted to act as a specific radiotracer of brain tumor tissue based on the low uptake by normal brain tissue compared with ^18^F-FDG and has proven specificity to detect gliomas [15]. No significant relationship between ^18^F-FAMT uptake and WHO grade of tumor was found in the first series of 15 glioma cases [21]. The current study has now demonstrated significantly different ^18^F-FAMT uptake in gliomas of various histologies and grades compared to ^18^F-FDG.

Recently, ^11^C-MET PET has become the most commonly used amino acid imaging modality for gliomas, although use is restricted to PET centers with an in-house cyclotron facility. ^11^C-MET PET is useful for detecting and delineating gliomas [5, 6, 25–28]. ^11^C-MET uptake shows positive correlation with astrocytoma grade (II/IV and III/IV) [5, 27]. However, oligodendroglioma, which is a low-grade tumor, may show higher uptake of ^11^C-MET than diffuse astrocytoma (WHO grade II) [5].^18^F-FAMT tracer was developed on the basis of the known accumulation in brain tumor tissue of L-3-[^123^I]iodo-α-methyl tyrosine [21]. Uptake of L-3-[^123^I]iodo-α-methyl tyrosine and of ^11^C-MET involves almost the same transport mechanism, system L, which is a Na-independent amino acid transport system, in cultured glioma cell lines [29]. In fact, L-3-[^123^I]iodo-α-methyl tyrosine single photon emission computed tomography and ^11^C-MET PET have equivalent clinical value in the diagnostic evaluation of glioma [29, 30]. Therefore, ^18^F-FAMT PET imaging is likely to have similar characteristics to ^11^C-MET PET imaging for glioma diagnosis. However, the cell transport systems of ^18^F-FAMT and ^11^C-MET may be different. L-type amino acid transporter 1 (LAT1) is a major route for the transport of large neutral amino acids, including L-tyrosine, L-leucine, and L-methionine, through the plasma membrane. LAT1 is essential in tumor growth and is widely expressed in primary human cancers as well as gliomas [31–33]. Recent findings have proved that ^18^F-FAMT is highly selective for LAT1 because of its α-methyl moiety [34], which suggests that the tumor imaging sensitivity and specificity of ^18^F-FAMT PET and ^11^C-MET PET may have subtle differences. Recently, another ^18^F-labeled amino acid tracer, ^18^F-FET, has been shown to be useful for PET diagnosis of glioma [8–10, 35]. ^18^F-FET PET has high T/N ratio and better contrast in all gliomas compared to ^18^F-FDG PET, similar to our findings for ^18^F-FAMT PET, and is a clinically valuable PET tracer for imaging of gliomas [8–10, 35]. ^18^F-FET was clearly proved to be transported through both LAT1 and LAT2, with less selectivity for LAT1 than ^18^F-FAMT [34]. However, a more recent study suggested that trapping of ^18^F-FET within the cells is caused by the asymmetry of its intra- and extracellular recognition by LAT1 [36]. Therefore, ^18^F-FET and ^18^F-FAMT have similar characteristics as ^18^F-based brain tumor imaging tracers, but with structural differences and different biological activities. Standard ^18^F-FET summation image analysis of the 20–40 min time frame revealed mean maximum tumor-to-background ratio (TBR_max_) of 2.1 in LGGs and significantly higher TBR_max_ of 3.3 in HGGs (*p* < 0.001) [37]. ROC analyses revealed a cutoff value of TBR_max_ 2.7 for the differentiation between LGGs and HGGs in the conventional 20–40 min summation images (sensitivity 66.7%, specificity 77.9%, accuracy 70.4%) [37]. In our series, ROC analysis for differentiation between HGGs and LGGs yielded an optimal cutoff value of 3.37 for the T/N ratio of ^18^F-FAMT (sensitivity 81%, specificity 67%, accuracy 76%) The cutoff value is higher than for ^18^F-FET PET, but the accuracy of ^18^F-FAMT uptake may be considered equivalent. Further study is required for comparison of the imaging characteristics of ^18^F-FET PET and ^18^F-FAMT PET for the diagnosis of glioma.

MIB-1 LI is considered to be an indicator of the simple cell proliferation rate. In contrast, WHO grade is a direct index of the malignancy grade, based on the consideration of various pathological factors, including the presence of necrosis, nuclear polymorphism, microvascular proliferation, mitotic activity, etc. The present investigation found that the T/N ratio of ^18^F-FAMT PET was not correlated, but the T/N ratio of ^18^F-FDG PET was correlated with MIB-1 LI in all gliomas. The increase in ^18^F-FAMT uptake does not necessarily indicate high cell proliferation activity. Comparisons of the T/N ratios of ^11^C-MET PET and the MIB-1 LI have found a significant correlation in diffuse astrocytoma but not in oligodendroglial tumor [5, 26, 28]. In our cohort, the ratio of diffuse astrocytoma was small, and the larger ratio of oligodendroglial tumor may have affected our results suggesting the T/N ratio of ^18^F-FAMT PET was not correlated with MIB-1 LI in all gliomas.

The glucose metabolic rate is highest in the brain parenchyma compared to the other organs of the body. Consequently, ^18^F-FDG is less effective as a tracer for the diagnostic imaging of brain tumor. Therefore, novel non-^18^F-FDG brain tumor radiotracers have been intensively researched in the past decade [2]. Multiple studies have compared brain tumor imaging with radiolabeled amino acids and ^18^F-FDG with the general finding that amino acids are more sensitive than ^18^F-FDG to detect brain tumors [12, 38–47]. Amino acids provide higher tumor-normal brain contrast and are better suited to delineate the tumor extent, to differentiate tumor recurrence from treatment-related changes, and to assess treatment response. Whether ^18^F-FDG or amino acids is the better choice for grading and prognosis remains controversial [48]. In our study, the T/N ratios of ^18^F-FAMT PET and ^18^F-FDG PET in the ROC analysis were almost equivalent for the differential diagnosis of tumor grade. ^18^F-FAMT uptake in the normal brain parenchyma was 0.94 (median SUV) in our series, lower than that of ^18^F-FDG, and almost the same as that of ^11^C-MET [27] and ^18^F-FET [8]. ^18^F-FAMT PET provided clearer imaging with higher T/N ratio and better contrast in all gliomas compared to ^18^F-FDG PET. Delineation of tumor extent and definition of the optimal site for biopsy are well-known and important advantages of amino acid PET at initial evaluation of brain tumors [38–40, 45]. In this study, the difference in T/N ratio between ^18^F-FAMT PET and ^18^F-FDG PET was significant. We are interested in whether ^18^F-FAMT PET can provide valuable data for the decisions concerning evaluation of true tumor size, extent of tumor excision range, and identification of the optimal site for biopsy. Further study will be necessary for these investigations.

More reliable grading may be possible with dynamic ^18^F-FET PET, since this tracer exhibits differences in the time-activity curves of tracer uptake depending on tumor grade [34]. HGGs are characterized by an early peak around 10–15 min after injection followed by a decrease of ^18^F-FET uptake. In contrast, LGGs typically exhibit delayed and steadily increasing tracer uptake [49]. The differential kinetics of tracer uptake in HGGs and LGGs appear to be a special property of ^18^F-FET because such differences were not observed with ^11^C-MET or L-6-[^18^F]fluoro-3,4-dihydroxyphenylalnine [11, 50]. Therefore, dynamic study with ^18^F-labeled tracer may be useful as an indicator of tumor grade. Further dynamic study using ^18^F-FAMT PET will be necessary in the future.

There were limitations to the present study. This study was based on relatively strict pathological and grading differentiations in astrocytomas and oligodendroglial tumors. Therefore, some pathological categories included a relatively small number of samples. Furthermore, simultaneous ^18^F-FAMT PET and ^18^F-FDG PET imaging is the ideal method of comparison. Since both tracers are labeled with fluorine, the tracer half-life requires a suitable interval between these PET studies. In these 38 cases, the interval between ^18^F-FAMT PET and ^18^F-FDG PET studies ranged from 1 to 38 days, and the median was 5 days. Recently, accumulation of ^18^F-FAMT was reported to be strongly correlated with the expression of LAT1 in cancers [34]. However, correlation of ^18^F-FAMT transport and LAT1 expression was not examined in this study. A further study will be needed to investigate the mechanism of ^18^F-FAMT accumulations in gliomas. ^18^F-FAMT is a new radiotracer for brain tumor imaging. More experience with cases of gliomas or other brain tumors is needed. A comparative study with radiotracers other than ^18^F-FDG is also needed to clarify the diagnostic utility of ^18^F-FAMT PET.

## Conclusions

^18^F-FAMT is a useful radiotracer for the preoperative evaluation of tumor malignancy. ^18^F-FAMT PET provides clearer imaging with higher T/N ratio and better contrast compared to ^18^F-FDG PET in all gliomas. Therefore, ^18^F-FAMT is a useful radiotracer for the preoperative visualization of gliomas.

## Abbreviations

AUC: Area under the curve
CI: Confidence interval
C-MET: L-[methyl-^11^C]methionine
CT: Computed tomography
F-FAMT: Fluorine-18α-methyltyrosine
F-FDG: Fluorine-18 fluorodeoxyglucose
F-FET: *O*-(2-[^18^F]fluoroethyl)-L-tyrosine
HGG: High-grade glioma
LAT1: L-type amino acid transporter 1
LGG: Low-grade glioma
MIB-1 LI: MIB-1 labeling index
NPV: Negative predictive value
PET: Positron emission tomography
PPV: Positive predictive value
ROC: Receiver operating characteristic
ROI: Region of interest
SUV: Standardized uptake value
SUV_max_: Maximum SUV
T/N ratio: Tumor to normal brain ratio
TBR_max_: Maximum tumor-to-background ratio
WHO: World Health Organization
